# Silver(I) Complexes of the Pharmaceutical Agents Metronidazole and 4-Hydroxymethylpyridine: Comparison of Cytotoxic Profile for Potential Clinical Application

**DOI:** 10.3390/molecules24101949

**Published:** 2019-05-21

**Authors:** Lidia Radko, Sylwia Stypuła-Trębas, Andrzej Posyniak, Dominik Żyro, Justyn Ochocki

**Affiliations:** 1 Department of Pharmacology and Toxicology, National Veterinary Research Institute, Partyzantów 57, 24-100 Puławy, Poland; lidia@piwet.pulawy.pl (L.R.); sylwia@piwet.pulawy.pl (S.S.-T.); aposyniak@piwet.pulawy.pl (A.P.); 2 Department of Bioinorganic Chemistry, Chair of Medicinal Chemistry, Medical University of Lodz, Muszyńskiego 1, 90-151 Łódź, Poland; dominik.zyro@umed.lodz.pl

**Keywords:** synthesis, silver(I) complexes, cytotoxicity, Balb/c 3T3 cell line, HepG2 cell line

## Abstract

In previous papers, we have reported on the high antifungal and significant antibacterial activity against Gram-positive and Gram-negative bacteria of the water-soluble silver(I) complexes of metronidazole and derivatives of pyridine compared to silver nitrate. In the present study, the cytotoxic activity of the silver(I) complexes of metronidazole and 4-hydroxymethylpyridine was compared with that of silver nitrate. Metronidazole and 4-hydroxymethylpyridine were investigated using Balb/c 3T3 and HepG2 cell lines in order to evaluate the potential clinical application of silver(I) complexes. The cells were exposed for 72 h to compounds at eight concentrations. The cytotoxic concentrations (IC_50_) of the study compounds were assessed within four biochemical endpoints: mitochondrial activity, lysosomal activity, cellular membrane integrity, and total protein content. The investigated silver(I) complexes displayed comparable cytotoxicity to that of silver nitrate used in clinics. Mean cytotoxic concentrations calculated for investigated silver(I) complexes from concentration–response curves ranged from 2.13 to 26.5 µM. HepG2 cells were less sensitive to the tested complexes compared to fibroblasts (Balb/c 3T3). However, the most affected endpoint for HepG2 cells was cellular membrane damage. The cytotoxicity of both silver complexes was comparable for Balb/c 3T3 cells. The cytotoxic potential of the new silver(I) compounds compared to that of silver nitrate used in medicine indicates that they are safe and could be used in clinical practice. The presented results are yet more stimulating to further studies that evaluate the therapeutic use of silver complexes.

## 1. Introduction

Research efforts targeting the development of new chemotherapeutic agents play an important role in medicine. High hopes of researchers are associated with silver ions. Silver has been used since ancient times for the purpose of wound healing and is a known antimicrobial agent that exhibits increased antimicrobial action. When silver comes in contact with microorganisms, there is immediate distortion of the cell wall, which later leads to the death of these organisms. Silver was proved to influence the metabolic behavior of bacteria, viruses, and eukaryotic microorganisms. It has been suggested that silver(I) ions modify their pathogenic activity through interaction with microbial electron transport systems, cell membranes, and the DNA-binding machinery. Silver exhibits broad-spectrum activity and a lower propensity to induce microbial resistance than conventional antibiotics. Furthermore, the antimicrobial action of silver can be potentiated by its combination with other antimicrobial agents [1,2,3]. The method for the synthesis of the silver complex with 4-hydroxymethylpyridine is the invention reported in a European patent specification [4] and has been published [5].

Ag(I) complexes show selective cytotoxicity toward various types of cells, and this is dependent on the type of ligand linked to the silver(I) ions. This dependency is probably related to the stability of the complexes and the hydrophilicity-lipophilicity of the complexes formed by the type of the ligand [6,7,8]. It is noteworthy to mention that the nitrates present in silver nitrate are toxic to the human body and can cause blue and/or bluish grey pigmentation, which is known as argyria [9]. The goal of many studies was to find out whether the toxicity of AgNO_3_ in complexes is reduced by the synergic action caused by the presence of metronidazole or 4-hydroxymethylpyridine molecules [1,5]. 

Metronidazole and pyridine derivatives display a wide range of pharmacological action with special emphasis on their antiviral, antibacterial, antiproliferative, and antifungal activities [10,11,12,13]. 

Metronidazole is a well-known antibiotic drug used to treat certain parasitic and bacterial infections. This synthetic derivative of nitroimidazole is active against *Trichomonas vaginalis* and *Entamoeba histolytica* and against the anaerobic bacteria *Bacteroides* spp., *Fusobacterium* spp., *Eubacterium* spp., and *Clostridium* spp. However, the compound is inactive against aerobic bacteria and fungi. 

Derivatives of pyridine show many pharmacological properties; for example, they play crucial roles in physiological functions, especially as part of the nicotinamide adenine dinucleotides (NAD^+^/NADH) coenzyme. Synthetic pyridine derivatives are used as pharmaceutical agents, such as isoniazid (an antibiotic used for the treatment of tuberculosis), piroxicam (an anti-inflammatory drug used in arthritis), niacin (a form of vitamin B3, an essential human nutrient), or vitamin B6.

The primary site of silver accumulation and metabolism of the drug is the liver, and it is therefore relevant that a number of in vitro investigations have focused on this potential target organ.

Investigation of in vitro toxicity is very important in many contexts. In vitro systems are used principally for screening and ranking chemicals and for generating more comprehensive toxicological profiles [14,15,16]. They are also of potential use for studying local or tissue effects and can target specific effects. Numerous suitable methods, each one with advantages and limitations, have been developed during the last years to evaluate cytotoxicity [17]. The combined use of four methods which estimate metabolic and lysosomal activity, membrane integrity, and proliferation is at present the most practical approach for evaluating basic cellular structures and functions [18,19,20,21,22]. The functioning opinion is that the assay selection is the most important factor governing the uniform quality of the data; however, the origin of the cell lines used is also of great importance [23]. At present, most in vitro studies are performed on permanent cell lines [24,25,26,27].

The aim of this study was to determine a cytotoxic potential of the silver complexes, namely, the silver(I) complex of metronidazole and the silver(I) complex of 4-hydroxymethylpyridine, as well as the free ligands metronidazole, 4-hydroxymethylpyridine, and silver nitrate. Additionally, the potential of cytotoxicity for the above-mentioned silver complexes was compared with the cytotoxic potential of silver nitrate used in clinics. Metronidazole, 4-hydroxymethylpyridine, and silver nitrate were also presented as the comparative criterion. We investigated their cytotoxicity effects on non-metabolizing and metabolizing, Balb/c 3T3 and HepG2 cell lines, respectively. 

The mouse fibroblast Balb/c 3T3 cells are the most frequently used cell line to screen the general toxicity of chemicals [19]. The HepG2 cell line is widely used for in vitro studies. It mostly expresses drug-metabolizing enzymes and is also widely used as a cellular experimental model in pharmaceutical studies for the production of new drugs and to gain insights into drug metabolism, including enzyme drug’s inhibition or induction potential [28,29].

To get better insight into the studied chemical’s mode of toxic action, four assays were used to assess various biochemical endpoints—mitochondrial activity [21], lysosomal activity [19], total protein content [20], and membrane integrity [22]. The half-maximal inhibitory (cytotoxicity) concentration 50 (IC_50_) values for study compounds were calculated. 

## 2. Results and Discussion

### 2.1. General Aspects

Both the synthesis and crystal structures of the silver(I) complexes of metronidazole and 4-hydroxymethylpyridine are known [1,5]. The silver(I) coordination compound of metronidazole (Met) is in the form of monomer [Ag(Met)_2_NO_3_]. The molecular structure of the complex was provided by X-ray single-crystal crystallography [1]. The silver(I) complex of 4-hydroxymethylpyridine (4-CH_2_OHpy)_2_ with the general formula [Ag(4-CH_2_OHpy)_2_]NO_3_ consists of one nitrate anion and one silver ion coordinated by two 4-hydroxymethylpyridine ligands [5]. The structural characterization (IR, ^1^H NMR, crystal X-ray diffraction analysis) and antimicrobial activity of these complexes were reported previously [1,5]. Herein, we report the synthesis of the complexes with some modification. 

### 2.2. Synthesis of [(Metronidazole)_2_AgNO_3_]

**Scheme 1:**
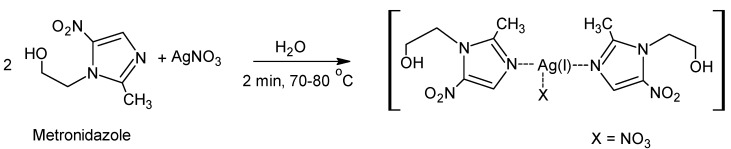
Synthesis of silver(I) complex of metronidazole.

The synthesis of the complex was performed earlier [1]. However, in the synthetic procedure in the present study, water was used as a solvent instead of a water/ethanol solution. The silver(I) complex of metronidazole was synthesized in an easy one-step process through the reaction of AgNO_3_ with metronidazole (1:2) (Scheme 1) in water. The complex was obtained with good yield and purity (see the Materials and Methods Section).

### 2.3. Synthesis of [(4-hydroxymethylpyridine)_2_Ag]NO_3_

**Scheme 2:**
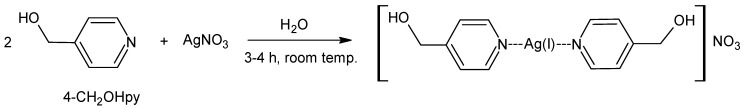
Synthesis of silver(I) complex of 4-hydoxymethylpyridine.

The synthesis of the silver complex of 4-hydroxymethylpyridine was also performed earlier [5]. Nevertheless, in the present study, water was used as a solvent instead of a water/ethanol solution. The silver(I) complex of 4-hydroxymethylpyridine was synthesized in an easy process through the reaction of AgNO_3_ with 4-hydroxymethylpyridine (1:2) (Scheme 2) in water. The complex was obtained with good yield and purity (see the Materials and Methods Section).

### 2.4. Cytotoxic Activity

**Figure 1:**
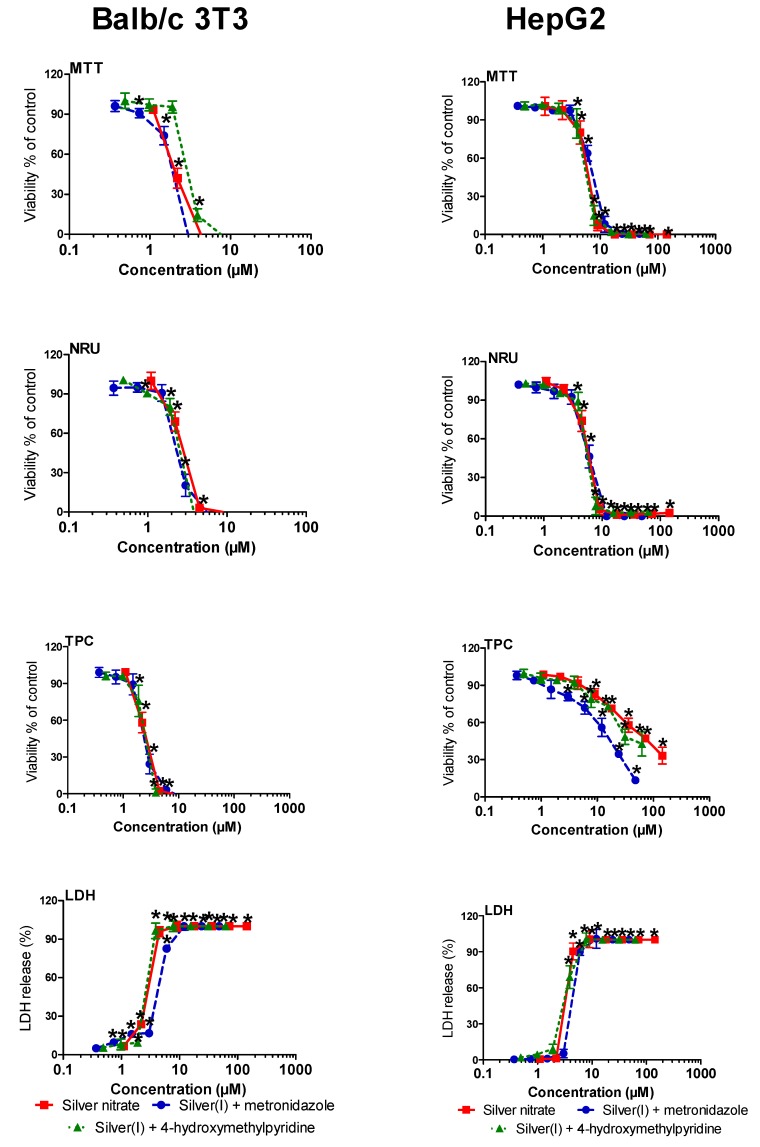
Concentration-dependent response curves for study compounds assessed by MTT, neutral red uptake (NRU), total protein content (TPC) and leakage lactate dehydrogenase (LDH) assays on Balb/c 3T3 and HepG2 cells. The results are expressed as the mean ± SD of three independent experiments. * *p* ≤ 0.05 in comparison with negative control (0.1% DMSO).

The cytotoxic potential of silver(I) complexes of metronidazole and 4-hydroxymethylpyridine as well as metronidazole, 4-hydroxymethylpyridine, and silver nitrate on Balb/c 3T3 and HepG2 cells was assessed by decreased viability of the cells within four biochemical endpoints: mitochondrial activity and lysosomal activity (MTT assays, reducing the tetrazolium dye MTT 3-(4,5-dimethylthiazol-2-yl)-2,5-diphenyltetrazolium bromide, and neutral red uptake (NRU) assays), total protein content (TPC assay), and cellular membrane integrity (leakage lactate dehydrogenase (LDH) assay) after 72 h exposition. Data obtained from the measurement of the optical density in these assays were transformed to percentages in relation to the control group, considered to be 100% for the MTT, NRU, and TPC assays and 0% for the LDH assay (Figure 1).

The half-maximal inhibitory (cytotoxicity) concentration (IC_50_) values were defined as the compound concentrations that reduced absorbance to 50% of control values. The cytotoxicity concentrations (IC_50_) are presented in Table 1. The values depended on the compound concentration, assay, and cell line used.

The drug non-metabolizing Balb/c 3T3 cells were more sensitive than the drug metabolizing HepG2 cells to the action of the silver(I) complexes of metronidazole and 4-hydroxymethylpyridine. 

The silver(I) complexes of metronidazole significantly (*p* ≤ 0.05) inhibited mitochondrial activity and disintegration of the cellular membrane of Balb/c 3T3 cells starting from the concentration of 0.74 µM (Figure 1, Balb/c 3T3: MTT, LDH). At the concentration of 3.0 µM, inhibited lysosomal activity and decreased total protein content in cell culture were observed (Figure 1, Balb/c 3T3: NRU, TPC). In the case of HepG2 cells, the same concentration (3.0 µM) also decreases total protein content in the cell cultures (Figure 1, HepG2: TPC). At the higher concentration of 6.0 µM, the significant (*p* ≤ 0.05) effects in other used endpoints were recorded (Figure 1, HepG2: MTT, NRU, LDH).

The silver(I) complex of 4-hydroxymethylpyridine significantly (*p* ≤ 0.05) inhibited lysosomal activity and disintegration of the cellular membrane of Balb/c 3T3 cells starting from the concentration of 0.98 µM (Figure 1, Balb/c 3T3: NRU, LDH). The decrease of total protein content in Balb/c 3T3 cell culture and inhibition of mitochondrial activity of the cells at concentrations of 1.9 and 3.8 µM were observed, respectively (Figure 1, Balb/c 3T3: TPC, MTT). In the case of HepG2 cells, at the concentration of 3.8 μM, the significant (*p* ≤ 0.05) inhibited mitochondrial and lysosomal activity and disintegration of the cellular membrane in the cell were observed (Figure 1, HepG2: MTT, NRU, LDH). At the concentration of 7.6 µM, decreased total protein content in the cell cultures was observed (Figure 1, HepG2: TPC).

The IC_50_ values for silver(I) complexes of metronidazole in the MTT, NRU, and TPC assays (≈2.17 µM) were two-fold lower than in the LDH assay (4.59 µM) in Balb/c 3T3 cells (Table 1). In the case of HepG2 cells, the IC_50_ values were the lowest in the LDH assay (4.61 µM) followed by the NRU and MTT assays and the TPC assay (Table 1). The highest IC_50_ value was in the TPC assay (15.17 µM) (Table 1). This value was the lowest in comparison with silver(I) complex of 4-hydroxymethylpyridine or silver nitrate (Table 1).

The IC_50_ value for the silver(I) complex of 4-hydroxymethylpyridine in all used assays was ≈3.0 µM in Balb/c 3T3 cells (Table 1). In the case of HepG2 cells, the IC_50_ values were the lowest in the LDH assay (3.50 µM) followed by the NRU and MTT assays (Table 1). The highest IC_50_ value was in the TPC assay (26.5 µM) (Table 1). This value was lower compared to that of silver nitrate in HepG2 cells (Table 1).

IC_50_ for the silver(I) complex of 4-hydroxymethylpyridine was 2.44 µM on B16 cells [3]. This value was lower than cytotoxic concentrations obtained for Balb/c 3T3 and HepG2 cells in our study. Another study showed that heteroleptic silver(I) complexes were excellent as cancer cell growth inhibitors of the breast (MCF-7), cervical (HeLa), epithelioma (Hep-2) and hepatoma (HepG2) cells, and normal human dermal fibroblasts (NHDF) [30]. Their IC_50_ values for HepG2 cells ranged from 7.38 to 8.48 µM [30]. 

It is worth noting that IC_50_ values for silver(I) complexes were lower in MTT, NRU, and TPC assays in Balb/c 3T3 cells compared to values obtained in HepG2 cells (Table 1). The tested silver(I) complexes were found to have relatively lower toxicity to the metabolized cell line, HepG2 (human hepatoma). This action was especially seen based on the TPC assay because it did not cause the detachment of the HepG2 cells. HepG2 cells have been shown to express cytochrome P450 activities and thus biotransformation activity. However, these activities are low in comparison with the human liver [28,29]. This impact of silver(I) complexes on HepG2 is not clear at this stage of the study and requires further research.

Moreover, the study silver(I) complexes were active against Gram-negative and Gram-positive strains, namely, *Pseudomonas aeruginosa* ATCC 15442, *Escherichia coli* ATCC 25922, *Proteus hauseri* ATCC 13315, *Staphylococcus aureus* ATCC 6538, and *Staphylococcus epidermidis* ATCC 12228. Their antibacterial potency was higher than that of the referenced drug AgNO_3_ [1,5]. 

The toxicity of silver nitrate has been investigated in various cell types [31,32]. We found that Balb/c 3T3 cells were more sensitive than HepG2 cells to the action of silver nitrate, similar to the study silver(I) complexes of metronidazole and 4-hydroxymethylpyridine. Significant (*p* ≤ 0.05) effects in all used endpoints in Balb/c 3T3 cells were observed starting from the concentration of 2.2 μM (Figure 1, Balb/c 3T3: MTT, NRU, TPC, LDH). In the case of HepG2 cells, the significantly (*p* ≤ 0.05) inhibited mitochondrial and lysosomal activities, as well as the disintegration of cellular membranes at the concentration 4.4 μM, were recorded (Figure 1, HepG2: MTT, NRU, LDH). At the higher concentration of 8.8 μM, silver nitrate decreased total protein content in the cell cultures (Figure 1, HepG2: TPC). A previous study showed that silver nitrate was not cytotoxic in the concentration range from 1 to 10 µM in murine melanoma cell line (B16F10) and murine melanocyte cell line (Melan-a) during 72 h incubation [33].

The mean cytotoxic concentrations (IC_50_) calculated for silver nitrate from concentration–response curves for Balb/c 3T3 and HepG2 cells were in the range of 2.13–3.12 µM and 3.47–60.3 µM, respectively (Table 1). The IC_50_ values of the compound for Balb/c 3T3 cells were lower than for HepG2 cells (Table 1). The highest value, a 20-fold difference, was shown between IC_50_ values in the TPC assay for both cell lines (Table 1). The silver nitrate did not cause the detachment of the metabolized cell line, HepG2 cells, based on the total protein content assay.

Recent studies have demonstrated that silver nitrate cytotoxicity depends on the type of cell lines. The cytotoxicity of silver nitrate has been studied on OVCAR-3 (ovarian), MB157 (breast), and HeLa (cervical) cell lines for 72 h. The inhibitory effects of silver nitrate (IC_50_ value) obtained were 35 µM, 5 µM, and 50 µM, respectively, for 72 h. Silver nitrate has been observed to be the most effective in the MB157 cell line compared to OVCAR-3 and HeLa cell lines [31]. The IC_50_ value of silver nitrate was 13.5 µM in A549 (lung) cells after 72 h exposure [34]. This means that silver nitrate is more effective on A549 cells compared to HeLa and OVCAR-3 cells. The higher concentrations of silver nitrate (1.5%) and longer exposure time (72 h) on NIH 3T3 fibroblasts show toxic action of the compound [32]. The IC_50_ values for silver nitrate on the murine melanoma (B16) cell line was 9.74 µM after 72 h incubation [3]. The shorter time exposure caused lower cytotoxicity of the compound. The IC_50_ values for silver nitrate on H-ras 5RP7 cells and NIH/3T3 cells were 6.75 µM and 12.3 µM, respectively, after 24 h exposure [35]. The IC_50_ values for AgNO_3_ after 24 h exposure of murine fibroblasts (L929) at MTT and NRU assay were 3.57 µM and 2.55 µM, respectively [36]. Silver nitrate is a strong inhibitor (IC_50_ value of 2.89 µM Ag) of HeLa cell lines [37].

Our study results showed that there was a higher cytotoxicity of silver nitrate on Balb/c 3T3 and HepG2 cell lines compared to the results obtained on other cell lines by other authors.

Metronidazole and 4-hydroxymethylpyridine were found to be poorly cytotoxic. The same observation was reported in B16 and 10T1/2 cell lines [3]. In both cell lines, the viability of the cells was mostly greater than 50% for metronidazole and 4-hydroxymethylpyridine even at the highest concentrations used (Figures S2 and S3, Supplementary Materials). However, metronidazole and 4-hydroxymethylpyridine significantly (*p* ≤ 0.05) inhibited the mitochondrial activity of Balb/c 3T3 cells starting from the concentrations of 36 µM and 13.6 µM, respectively (Figure S2, Supplementary Materials, MTT). Additionally, 4-hydroxymethylpyridine significantly (*p* ≤ 0.05) inhibited the lysosomal activity of the cells and decreased total protein content in the cell cultures at concentrations of 7.0 µM and 57 µM, respectively (Figure S2, Supplementary Materials, NRU, TPC). In HepG2 cells, only metronidazole significantly (*p* ≤ 0.05) decreased total protein content in the cell cultures and inhibited lysosomal activity at concentrations of 8.8 µM and 146 μM, respectively (Figure S3, Supplementary Materials, TPC, NRU). The IC_50_ values were not calculated for metronidazole and 4-hydroxymethylpyridine in both cellular models (up to the highest concentrations of 227 μM and 146 μM, respectively) (Table 1).

Metronidazole was previously characterized as the compound with low cytotoxicity in HepG2 and FaO cells [14].

In the literature, there are few studies on the evaluation of the cytotoxic potential of metronidazole. It has been demonstrated that metronidazole at the concentration of 50 µg/mL decreased the viability of human fibroblasts (FMM1) by 40% after 72 h treatment [38]. Metronidazole was not cytotoxic at the concentration range from 2.5 to 250 µM in MCF7 cells, during 48 h incubation, but 30% inhibition was observed after exposure to 250 µM [39]. An earlier study indicated that a concentration of 84.8 µM inhibited the viability of IEC-6 cells only by 8% after 24 h exposure [40].

## 3. Materials and Methods 

### 3.1. Chemicals and Reagents

Silver complexes were synthesized according to the previously described procedure [1,5]. All solvents used were of reagent grade. Analytical standards of metronidazole (CAS: 443-48-1; M.W. 171.5 g/mol), 4-hydroxymethylpyridine (CAS: 586-95-8; molecular weight: 110 g/mol), and silver nitrate (CAS: 7761-88-8; molecular weight: 169 g/mol) were purchased from Sigma-Aldrich (Poznań, Poland). Triton X-100, dimethyl sulfoxide (DMSO), fetal bovine serum (FBS), bovine calf serum (BCS), neutral red dye (NR), Coomassie Brilliant Blue R-250 dye, 3-(4,5-dimethylthiazol-2-yl)-2,5-diphenyltetrazolium bromide (MTT), trypsin-EDTA, and antibiotic solution (10 000 U/mL of penicillin, 10 mg/mL of streptomycin) were purchased from Sigma-Aldrich (Poland). All other chemicals were purchased from commercial suppliers and were of the highest available purity. Melting points were determined with a Boethius apparatus (Franz Küstner Nachf. KG, HMK, Dresden, Germany) and have been uncorrected.

### 3.2. Synthetic Procedures

#### 3.2.1. Synthesis of [(Metronidazole)_2_AgNO_3_]

The synthesis was based on the procedure described elsewhere [1] with small modifications. Specifically, a 3.42 g portion (20 mmol) of metronidazole was added to a solution of AgNO_3_ (1.7 g, 10 mmol) in water (50 mL). The reaction mixture was stirred at 80 °C temperature for 2 min. After cooling to the ambient temperature, the resulting white crystalline precipitate (needles) (Figure S1, Supplementary Materials) of the complex was filtered off, washed with diethyl ether, and air dried. Molecular weight: 512,182 g/mol; yield: 4.19g (81.80%), Elemental analysis measured (calc.%): C 28.28 (28.14), H 3.09 (3.54), N 19.23 (19.14); melting point: 150–152 °C.

#### 3.2.2. Synthesis of [(4-hydroxymethylpyridine)_2_Ag]NO_3_

The synthesis was based on the procedure described elsewhere [5], with small modifications. Briefly, a 680 mg portion (4.0 mmol) of silver nitrate was added to a solution of 4-hydroxymethylpyridine (872 mg, 8 mmol) in water (20 mL). The mixture was vigorously stirred at room temperature for 4 h. The powder of impurities (gray) was filtered off. The solution was evaporated producing a slightly yellow precipitate. The crude product was purified by recrystallization in ethanol giving slightly yellow crystals. Molecular weight 388.126 g/mol; yield: 1.37 g (86.14%), Elemental analysis measured (calc.%): C 37.24 (37.13), H 3.45 (3.64), N 11.03 (10.83); melting point: 127–129 °C.

### 3.3. Cell Line Cultures 

The human hepatoma cell line (HepG2) was purchased from the American Type Culture Collection (ATCC HB-8065). These cells were cultured in Minimum Essential Medium Eagle (MEME) (ATCC). The murine fibroblast cell line (Balb/c 3T3 clone A31) (gift from Department of Swine Diseases of the National Veterinary Research Institute in Pulawy) was cultured in Dulbecco’s Modified Eagle’s Medium (DMEM) (ATCC). The media were supplemented with 10% BCS (Balb/c 3T3), 10% FBS (HepG2), 1% L-glutamine, and 1% antibiotic solution. The cells were maintained in 75 cm^2^ cell culture flasks (Nunc, Roskilde, Denmark) in a humidified incubator, NuAire (Plymouth, MN, USA) at 37 °C, in an atmosphere of 5% CO_2_. The medium was refreshed every two or three days and the cells were trypsinized by 0.25% trypsin–0.02% EDTA after reaching 70–80% confluence. Single cell suspensions were prepared and adjusted to a density of 2 × 105 cells/mL (HepG2) and 5 × 104 cells/mL (Balb/c 3T3). The cell suspension was transferred to 96-well plates (100 µL/well) and incubated for 24 h before the exposure to the study compounds.

### 3.4. Preparation of Compounds and Exposure

The concentration ranges of compounds were chosen according to their solubility and their plasma level. Each compound was dissolved in DMSO. The final concentration of DMSO was 0.1% in the medium. The same final concentrations of the solvent and the 1% Trition-X 100 solution were used as the negative and positive controls, respectively. The medium used for test solutions and in control preparation did not contain serum and antibiotics. All compound solutions in the medium were freshly prepared and protected from light. Each compound was tested in eight concentrations ranging from 0.37 to 48 µM (for the silver(I) complex of metronidazole), from 0.5 to 12.5 µM (for the silver(I) complex of 4-hydroxymethylpyridine), from 1.0 to 144 µM (for silver nitrate), from 1.0 to 146 µM (for metronidazole), and from 1.7 to 227 µM (for 4-hydroxymethylpyridine). The concentration range was selected on the basis of results from previous studies [3,14]. Each concentration was tested in six replicates with three independent experiments. Cytotoxicity was assessed after 72 h of exposure of the cells to the compounds. The medium was not changed during the incubation time.

### 3.5. Cytotoxicity Assessment

#### 3.5.1. MTT Assay

The metabolic activity of living cells was assessed by measuring the activity of dehydrogenases [21]. After incubation of the cells with the compounds, a 10 μL portion of the MTT solution (5 mg/ml in PBS) was added to each well of the 96-well plates and incubated. After 3 h, the MTT solution was removed and the intracellular formazan crystals were dissolved in 100 µl DMSO. The plate was shaken for 15 min at room temperature and transferred to a microplate reader (LabSystems Multiskan RC, Thermo Fisher Scientific, Waltham, MA, USA) to measure the absorbance at 570 nm, using blanks as a reference. Cytotoxicity was expressed as a percentage of the negative control (0.1% DMSO).

#### 3.5.2. NRU Assay

The assay, based on the staining of living cells by neutral red, was performed according to the protocol described by Borenfreund and Puerner [19]. After incubation, the medium containing the compound was removed and the cells were washed with PBS. Then, a 100 µL/well of NR solution (50 µg/mL) was added for 3 h. After that time, the cells were washed with PBS. The dye from viable cells was released by extraction with a mixture of acetic acid, ethanol, and water. After 10 min of shaking, the absorbance of the dissolved NR was measured at 540 nm in a microplate reader (LabSystems Multiskan RC, Thermo Fisher Scientific, Waltham, MA, USA) using a blank as a reference. Cytotoxicity was expressed as a percentage of the negative control (0.1% DMSO).

#### 3.5.3. TPC Assay

The assay was based upon staining total cellular protein (proliferation) [20]. After incubation, the medium containing the compound was removed and 100 µL of Coomassie Brilliant Blue R-250 dye were added to each well. The plate was shaken for 10 min. Then, the stain was removed and the cells were rinsed twice with 100 µL of washing solution (glacial acetic acid/ethanol/water). After that, a 100 µL portion of the desorbing solution (1 M potassium acetate) was added and plates were shaken again for 10 min. The absorbance was measured at 595 nm in a microplate reader (LabSystems Multiskan RC, Thermo Fisher Scientific, Waltham, MA, USA) using a blank as a reference. Cytotoxicity was expressed as a percentage of the negative control (0.1% DMSO).

#### 3.5.4. Leakage LDH Assay

The integrity of the plasma membrane was assessed using the test of lactate dehydrogenase (LDH) release [22], which was monitored using the commercially available Cytotoxicity Detection Kit (LDH) (Roche Diagnostics, Warsaw, Poland). The medium (100 µL/well) without cells was transferred into the corresponding wells of an optically clear 96-well flat bottom microplate and a 100 µl reaction mixture was added to each well. Then, the plates were incubated for 30 min at room temperature in darkness. After that time, a 50 µL/well 1 M HCl was added to stop the reaction. The absorbance was measured at 492 nm in a microplate reader (LabSystems Multiskan RC, Thermo Fisher Scientific, Waltham, MA, USA) using a blank as a reference. 

### 3.6. Statistical Analysis

The results of the tests were expressed as a mean ± SD. The experiments were performed in three independent repetitions. Statistical analysis was performed using GraphPad Prism 5 (San Diego, CA, USA). One-way analysis of variance (ANOVA) followed by Dunnett’s post-hoc test was applied. The 50% inhibition (cytotoxicity) concentrations (IC_50_) were derived from the dose–response curve plotted with the concentration of the compound (*X*-axis) vs. cell viability percent (*Y*-axis). The IC_50_ values were calculated by GraphPad Prism 5 software (San Diego, CA, USA). Statistical comparisons among IC_50_ values were performed by the analysis of variance (ANOVA) followed by the Tukey test. Values of *p* ≤ 0.05 were considered statistically significant.

## 4. Conclusions

In conclusion, we modified a concise and convenient route for the synthesis of the coordination silver(I) compounds of metronidazole and of 4-hydroxymethylpyridine. This is of interest in view of the biological importance of our experiments, which turned out to be very promising because they provide evidence for their practical application. The cytotoxicity was assessed against the non-metabolized cell line, Balb/c 3T3 (murine fibroblasts), and the metabolized cell line, HepG2 (human hepatoma). The tested silver(I) complexes and silver nitrate were found to have relatively higher toxicity to the non-metabolized cell line, Balb/c 3T3 (murine fibroblasts). The tested silver(I) complexes displayed comparable toxicity with AgNO_3_ in both cell lines. However, their antibacterial potency was higher than that of AgNO_3_, a drug which is used in clinical practices as shown in previous studies. The synergic activity of the silver(I) complexes of metronidazole and pyridine derivatives may have an important impact on the development of combination therapy, prophylaxis, and prevention of infections caused by bacteria, fungi, yeasts, or viruses. After all, the Credé procedure—the dropping of a silver nitrate solution into the eyes of newborn infants to prevent the development of gonorrhea ophthalmia—is widely used in medicine [41]. The cytotoxic potential of the new silver(I) compounds compared to that of silver nitrate used in clinics indicates that they are safe and could be used in clinical practice. 

## Figures and Tables

**Table 1:** Inhibitory concentrations (IC_50_, µM) determined in Balb/c 3T3 and HepG2 cells by MTT, NRU, TPC, and LDH assays after 72 h exposure to study compounds; data presented as mean (*n* = 3) ± SEM (standard error mean). The different superscripts (a, b, c) within a line indicate significant differences between the used assay (*p* ≤ 0.05).

	Balb/c 3T3				HepG2			
	MTT	NRU	TPC	LDH	MTT	NRU	TPC	LDH
**Silver(I) + metronidazole**	2.19 ± 0.29 ^a^	2.17 ± 0.57 ^a^	2.13 ± 0.51 ^a^	4.59 ± 0.08 ^a^	7.61 ± 1.07 ^a^	5.92 ± 1.13 ^ac^	15.2 ± 2.4 ^b^	4.61 ± 0.1 ^c^
**Silver(I) + 4-hydroxymethylpyridine**	3.37 ± 0.57 ^a^	3.22 ± 0.46 ^a^	3.35 ± 0.75 ^a^	3.07 ± 0.08 ^a^	6.53 ± 1.19 ^a^	5.95 ± 0.08 ^a^	26.5 ± 5.8 ^b^	3.50 ± 0.08 ^c^
**Silver nitrate**	2.13 ± 0.88 ^a^	2.94 ± 1.06 ^ab^	2.59 ± 1.30 ^ab^	3.12 ± 0.06 ^b^	6.48 ± 1.60 ^a^	6.18 ± 1.41 ^a^	60.3 ± 8.1 ^b^	3.47 ± 0.24 ^c^
**Metronidazole**	>146	>146	>146	>146	>146	>146	>146	>146
**4-hydroxymethylpyridine**	>227	>227	>227	>227	>227	>227	>227	>227

## Supplementary Materials

The following are available online, Figures S1–S3.
