# Supplementary material for: Silver(I) Complexes of the Pharmaceutical Agents Metronidazole and 4-Hydroxymethylpyridine: Comparison of Cytotoxic Profile for Potential Clinical Application
Source: Molecules. 2019 May 21;24(10):1949. doi: 10.3390/molecules24101949 (PMC6572996; doi:10.3390/molecules24101949)
Supplement: Supplementary file 1 [file molecules-24-01949-s001.pdf]

Article

Silver(I) Complexes of the Pharmaceutical Agents Metronidazole and 4-Hydroxymethylpyridine: Comparison of Cytotoxic Profile for Potential Clinical Application

Lidia Radko [1], Sylwia Stypuła-Trębas [1], Andrzej Posyniak [1], Dominik Żyro [2] and Justyn Ochocki [2,*]

[1] Department of Pharmacology and Toxicology, National Veterinary Research Institute, Partyzantów 57, 24-100 Puławy, Poland; lidia@piwet.pulawy.pl (L.R.); sylwia@piwet.pulawy.pl (S.S.-T.); aposyniak@piwet.pulawy.pl (A.P.)

[2] Department of Bioinorganic Chemistry, Chair of Medicinal Chemistry, Medical University of Lodz, Muszyńskiego 1, 90-151 Łódź, Poland; dominik.zyro@umed.lodz.pl

* Correspondence: justyn.ochocki@umed.lodz.pl; Tel.: +48-42-6779-220

Contents:

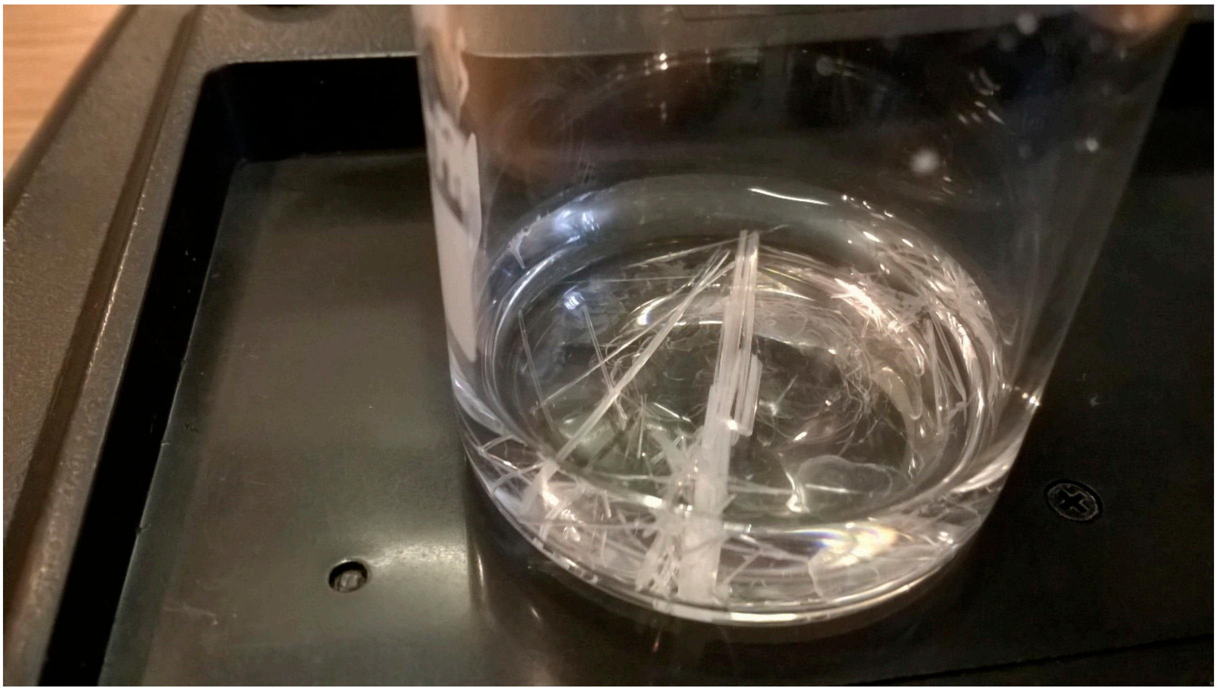

Figure S1. Photograph of silver(I) complex of metronidazole obtained as a result of one-step synthesis.

Balb/c 3T3

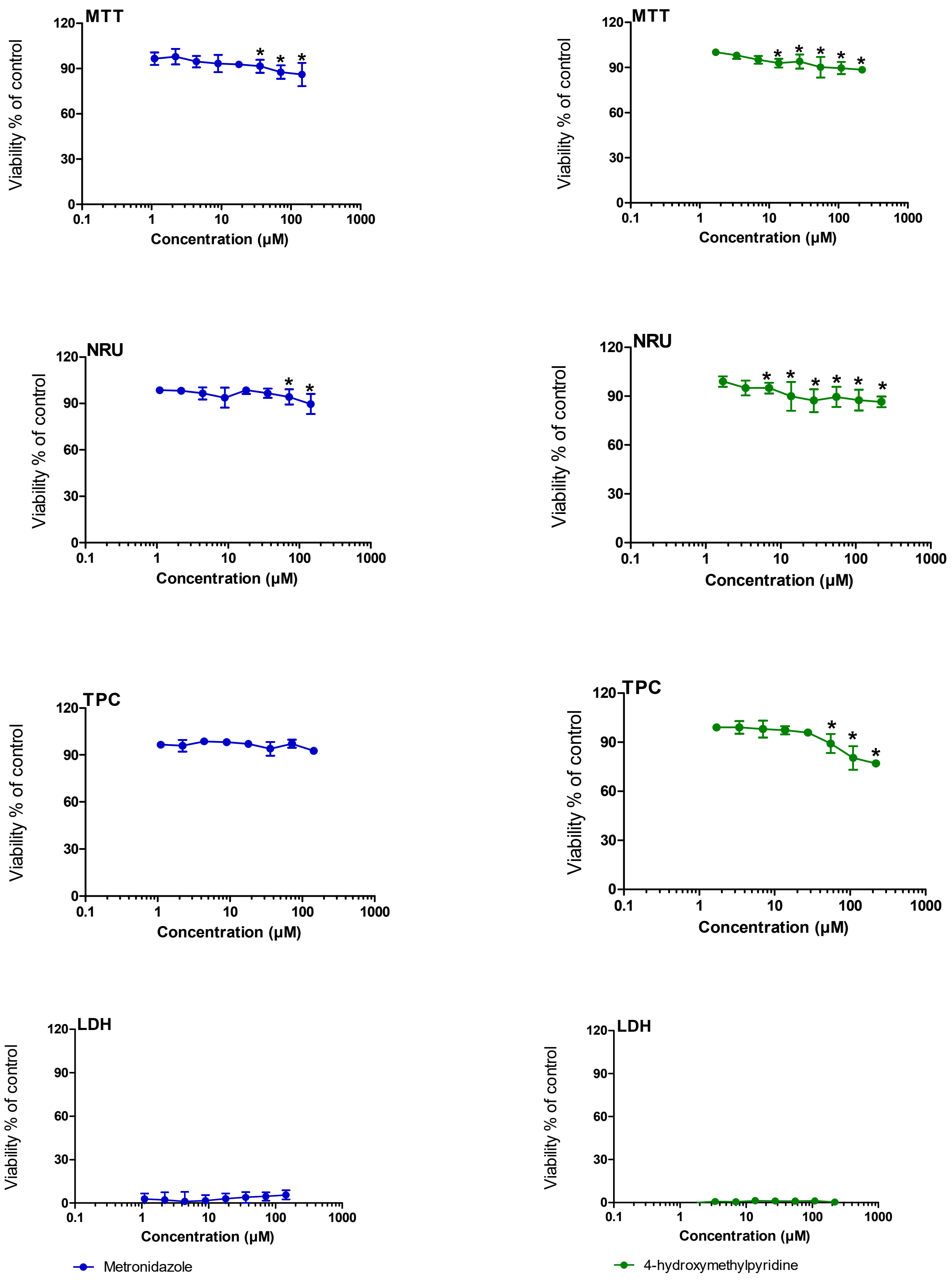

Figure S2. Concentration-dependent response curves for metronidazole and 4-hydroxymethylpyridine assessed by MTT, NRU, TPC, and LDH assays on Balb/c 3T3 cells. The results are expressed as the mean ± SD of three independent experiments. * $p \leq 0.05$ in comparison with negative control (0.1% DMSO).

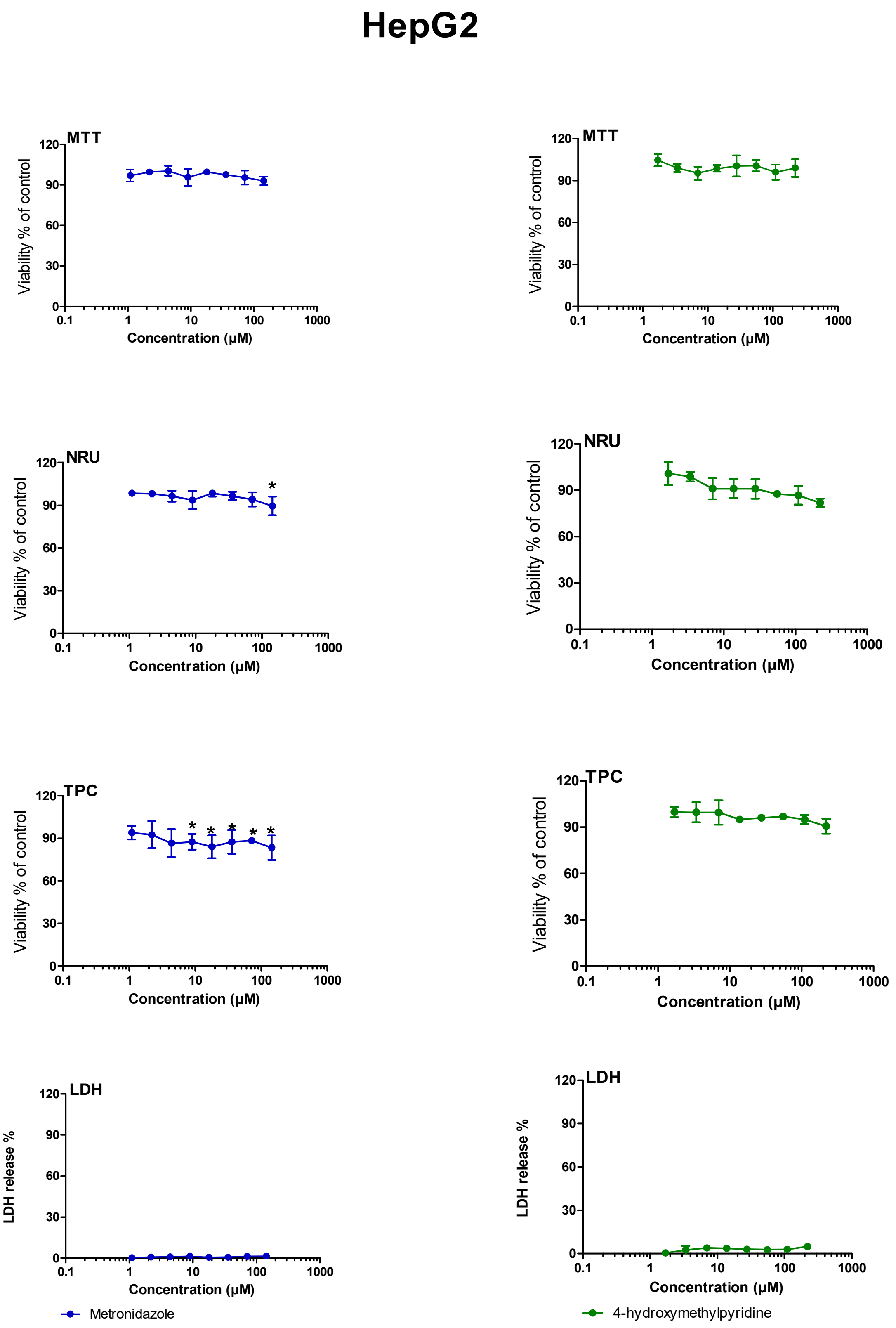

Figure S3. Concentration-dependent response curves for metronidazole and 4-hydroxymethylpyridine assessed by MTT, NRU, TPC, and LDH assays on HepG2 cells. The results are expressed as the mean ± SD of three independent experiments. * $p \leq 0.05$ in comparison with negative control (0.1% DMSO).
